# Supplementary material for: Detection of balance in the elderly under the influence of stress (DEPIE): A cross-sectional study protocol
Source: PLoS One. 2026 Jan 27;21(1):e0341744. doi: 10.1371/journal.pone.0341744 (PMC12843555; doi:10.1371/journal.pone.0341744)
Supplement: S2 File — (DOCX) [file pone.0341744.s002.docx]

| **Ethics** | | |  |
| --- | --- | --- | --- |
| Research ethics approval | 30 | Plans for seeking research ethics committee/institutional review board approval | 4, 11 |
| Protocol amendments | 31 | Plans for communicating important protocol modifications to relevant parties | 29 |
| Consent or assent | 32a | Who will obtain informed consent or assent from potential trial participants or authorized proxies, and how | 11-12 |
|  | 32b | Additional consent provisions for collection and use of participant data and biological specimens in ancillary studies, if applicable | Not Applicable |
| Confidentiality | 33 | How personal information about potential and enrolled participants will be collected, shared, and maintained in order to protect confidentiality before, during, and after the trial | 13 |
| Ancillary and post-trial care | 34 | Provisions, if any, for ancillary and post-trial care, and for compensation to those who suffer harm from trial participation | 13 |

*We strongly recommend reading this checklist in conjunction with the SPIRIT 2025 Explanation and Elaboration and the SPIRIT 2025 Expanded Checklist for important clarifications on all the items. We also recommend reading relevant SPIRIT extensions. See [www.consort-spirit.org](http://www.consort-spirit.org)

Citation: Chan A-W, Boutron I, Hopewell S, Moher D, Schulz KF, et al. SPIRIT 2025 statement: updated guideline for protocols of randomised trials. BMJ 2025;389:e081477. <https://dx.doi.org/10.1136/bmj-2024-081477>

© 2025 Chan A-W et al. This is an Open Access article distributed under the terms of the Creative Commons Attribution License (<https://creativecommons.org/licenses/by/4.0/>), which permits unrestricted use, distribution, and reproduction in any medium, provided the original work is properly cited.
