# Supplementary material for: Detection of balance in the elderly under the influence of stress (DEPIE): A cross-sectional study protocol
Source: PLoS One. 2026 Jan 27;21(1):e0341744. doi: 10.1371/journal.pone.0341744 (PMC12843555; doi:10.1371/journal.pone.0341744)
Supplement: Attachment — Submitted filename: Rebuttal_letter.pdf [file pone.0341744.s003.pdf]

Dear Editor and Reviewers,

We are very grateful to the editor and three reviewers who proposed several constructive and helpful suggestions.

Based on their comments, the present modifications should contribute to improving its quality. All major and minor comments have been considered, and we thank you for your careful reading.

Here are the point-by-point responses in the order in which the questions appeared in the comments.

Editor Comments:

Comments	Response
1. Study design and terminology Revise the description so that terminology is accurate and consistent. In particular, avoid describing the study as "double-blind," as this term applies to interventional designs, not cross-sectional observational studies. Also clarify what "analytical" means in this context, or use more standard terminology. State clearly that this is a cross-sectional, laboratory-based observational study with outcome assessors and/or data analysts masked where appropriate.	The design is now described as a "cross-sectional, laboratory-based observational study," and the term "double-blind" has been removed. Masking is specified only for outcome assessors and data analysts, and the term "analytical" is clarified as referring to comparisons between groups and conditions. These modifications directly address Reviewer 1’s comments in the Abstract and Methods sections.
2. Primary outcomes, statistical analysis, and sample size Define a single primary outcome (or a clearly prioritised set) and distinguish it from secondary and exploratory outcomes. Align the sample-size calculation with the primary outcome and intended statistical test, and justify the chosen effect size or acknowledge in the limitations that smaller effects may be underpowered. Provide a more structured statistical analysis plan, including handling of missing data and multiplicity control. Clarify the role of machine-learning analyses as exploratory and outline the basic framework (cross-validation, performance metrics, avoidance of data	We appreciate the reviewer's comments. Their suggestions and those made by the reviewers have been considered. Given that this study is part of a broad project with different objectives aimed at exploring the effect of emotional stress on balance in young and older adults, a priority set of primary outcomes has been defined to address this issue. The sample size was calculated based on the effect sizes described in the previous literature on the impact of stress on balance in both young and older adults. This ensures that the study has sufficient power to analyse the set of primary variables in the intended populations. This information has been added to the manuscript in the “Materials and methods” section.

leakage).	Furthermore, the statistical analysis has been revised and the necessary issues have been added to address it more comprehensively, including handling of missing data and multiplicity control. Revisions regarding this issue can be found in the “Statistical Analysis” section.<br><br>Finally, thank you for your valuable suggestion to clarify the exploratory nature of the data analysis. The paragraph has been revised accordingly, explicitly stating its primarily exploratory character and incorporating details on cross-validation, key performance metrics (accuracy, precision, recall, F1-score), and data leakage prevention through careful dataset partitioning and transformations applied exclusively within the training pipeline. To enhance paragraph readability, specific algorithms have been generalized to technique categories.
3. Inclusion criteria, consent, and risk Specify inclusion criteria beyond age: for example, language comprehension, corrected vision sufficient for image-based tasks, and ability to walk safely. Expand the informed-consent description (verbal explanation, time to consider, opportunity to ask questions). Revisit the risk statement: acknowledge potential transient distress from high-arousal images, specify stopping criteria, and describe debriefing and support options.	Inclusion criteria have been detailed to specify adequate comprehension of Spanish, corrected vision sufficient for the IAPS images, and safe ambulation in the laboratory. The informed consent description has been expanded to include verbal explanation, time to consider participation, opportunities to ask questions, and consultation with family or a trusted person if desired. The risk statement has been revised and explicitly described in the main manuscript, and both the original and translated versions of the Participant Information Sheet used at study entry are provided as supplementary material. These changes address the concerns raised by Reviewers 1, 2, and 3.
4. Data protection and GDPR Clarify how data flow from sensors to storage and analysis (local vs. vendor cloud, encryption, pseudonymisation, retention). Explain briefly how the procedures comply with GDPR, particularly if any data are transmitted or stored outside the EU.	Thank you for your valuable suggestion regarding data protection and GDPR compliance.<br><br>Data security breach risk minimized through pseudonymization (unique codes from outset), local storage (UAH servers/institutional cloud storage private folder, researcher access only; physical data under lock/key), and GDPR compliance (EU 2016/679, LOPDGDD 3/2018) - no identifiable personal data

	collected.<br><br>There is one device requiring special processing: Hexoskin data are stored on the Hexoskin server (AWS data centres, US West/East Coast). Some processing (HRV calculation) occurs there, as Hexoskin offers no EU-exclusive server option. However, local storage on computer (no server sync) is available, though this skips Hexoskin HRV processing.<br><br>In any case, these data are stored completely anonymously (no identifiable personal data collected), with ethics committee approval for identification during storage/processing. If the reviewer considers it necessary to expand this information in the manuscript, please let us know.
5. Alignment of measures with hypotheses and study flow Link each class of measure to the main hypotheses—a concise table mapping hypotheses to measures would help. Provide a clearer timeline or flow diagram, including approximate durations and the rationale for the fixed order of conditions. Clarify measurement scope (e.g., whether EMG is recorded during TUG and FRT).	We thank the editor for this helpful comment regarding the alignment between hypotheses, measures, and study flow. In the revised manuscript, we have clarified how each set of measures relates to the main objectives and hypotheses, and we have improved the description of the temporal sequence and study procedures. These clarifications, including a clearer presentation of the order of conditions, timings, and the scope of sensor-based recordings, address all the issues raised by the reviewers regarding study flow and measurement structure, and we believe they have substantially improved the clarity of the protocol.
6. Conclusion, data availability, and minor issues Reconsider statements in the Conclusion that may overstate what the study can demonstrate (e.g., the claim that results will "make it possible to take action before stressful situations arise"). Ensure the data-availability statement is internally consistent, indicating the planned repository, timing of release, and what will be shared. Correct minor inconsistencies (e.g., reference formatting) in line with	We have revised the Conclusion to remove overly strong statements and to focus more precisely on the neuromuscular mechanisms investigated and the results that can be obtained from the planned data. The data availability statement has been clarified, indicating the planned repository and timing linked to ClinicalTrials.gov, and the information provided to participants is fully documented both in the main manuscript and in the Participant Information Sheet supplied as supplementary material in its original Spanish version and in English translation. The reference list has also been reviewed and corrected to

journal style.	conform to Vancouver/PLOS ONE style, including the complete SENIAM citation and providing reference 27 in English. These modifications address the points raised by Reviewers 2 and 3.

Reviewer 1:

Comments	Response
Abstract: Double-blind is a feature of interventional designs (e.g., randomized controlled trials, not observational cross-sectional ones. If the study is analytical, it can compare two or more groups (e.g., exposed vs. non-exposed, etc), but it cannot be double-blind if cross sectional.	We thank the reviewer for this important clarification regarding study design terminology. We agree that the term "double-blind" is not appropriate for an observational cross-sectional protocol. This study is a cross-sectional, laboratory-based observational study conducted in a single session, in which participants are exposed to emotional stressors (high-arousal images) and their immediate neuromuscular and balance responses are observed. We have revised the Abstract and Methods accordingly and now specify that masking applies only to outcome assessors and data analysts, rather than describing the design as double-blind.
Line 210: The detailed inclusion criteria is to be provided.	We thank the reviewer for this helpful comment. We have now provided a more detailed description of the inclusion criteria in the "Participants" section. Specifically, we clarify that inclusion criteria comprise: ability to understand spoken and written Spanish sufficiently to follow the study information and instructions, corrected or uncorrected vision sufficient to view the image-based tasks, ability to walk safely over short distances in the laboratory setting with or without usual assistive devices, and willingness to provide written informed consent. Participants are included if they wish to take part voluntarily and do not present any of the specified exclusion criteria (illness or injury contraindicating muscle exertion, balance exercises and/or walking, or physical/mental illness contraindicating exposure to emotionally stressful stimuli). The manuscript has been revised accordingly in the "Participants"

	subsection of the Methods.
Line 216: More details of the sample size calculation are to be provided, e.g., one or two-tailed test, outcome variable, and effect size figure, test family, statistical test, type of power analysis etc.	We thank the reviewer for their comment. Following their suggestion, the requested elements have been added to the section entitled 'Participants' in the paragraph relating to sample size calculation.<br><br>In this regard, it should be noted that this study is part of a broader project in which one of the main objectives is to compare alterations in balance, center of mass displacement and muscle activation induced by emotional stressors in young and older adults. Given that several primary outcome variables are analyzed, the sample size was calculated based on the effect size reported in previous studies that have addressed the impact of stress on balance in these populations.
The study design is unclear. A proper flow chart to illustrate the study design from the beginning, participants, and how the participant is assigned, the exposed groups/intervention groups, duration/period, the assessment point, outcome etc is to be provided. Currently, in the absence of random allocation, the study appears to adopt a prospective cohort design.	We thank the reviewer for their comment. To improve clarity in the presentation of the study design, Figure 2 has been modified accordingly. We hope that this modified Figure 2, together with Figure 1 (SPIRIT diagram), will help to clarify the issues raised by the reviewer.
Line 270, Line 284, Line 293, Line 304, Line 321, Line 333: The language version of the scales is to be stated.	We appreciate this comment. We confirm that all neuropsychological and physical assessment tools described in the Methods (cognitive and physical assessment subsections) are administered in their validated Spanish versions or Spanish-adapted forms, as appropriate for our study population. In the revised manuscript, we now clarify in the "Baseline cognitive assessment" subsection that all neuropsychological tests are administered in Spanish, and in the "Baseline physical assessment" subsection that all physical performance tests are also administered in Spanish. As this applies uniformly to all listed instruments, the text has been updated to indicate explicitly that the

	assessments are conducted in Spanish.
Line 448-449: The focus is to be on the primary outcome and followed by secondary outcome and not vice versa.	We thank the reviewer for this important comment on the ranking of results. We agree that the primary variables constitute the core of the study.<br><br>However, we have maintained the current order of presentation due to a requirement of the experimental design: the secondary variables (HRV, RR, VAS) act as a manipulation check. In other words, it was essential to first verify that the stressor stimulus was effective at the psychophysiological level to subsequently attribute the changes in the primary variables to that stress. We have revised the text ('Statistical Analyses') to ensure that this logic is clear and that the primary result is not minimized.

Reviewer 2:

Comments	Response
Sample-size clarification.<br><br>The sample-size calculation assumes a medium effect size (f = 0.25) with α = 0.05 and 80% power, leading to 27 participants per group. While reasonable for detecting medium or larger effects in a 2×2 mixed ANOVA framework, smaller interaction effects are common in clinical and rehabilitation research. A brief justification of the chosen effect size based on prior literature, or mention in the limitations that smaller effects may be underpowered, would enhance transparency without requiring changes to the study plan.	We are very grateful to the reviewer for their comment. Following their recommendations and the comments of reviewer 1, a brief justification for the chosen effect size has been added to the section entitled 'Participants' in the paragraph relating to sample size calculation.<br><br>In this regard, it should be noted that this study is part of a broader project in which one of the main objectives is to compare alterations in balance, center of mass displacement and muscle activation induced by emotional stressors in young and older adults. Given that several primary outcome variables are analyzed, the sample size was calculated based on the effect size reported in previous studies that have addressed the impact of stress on balance in these populations.

Cognitive assessment procedure.<br><br>The cognitive evaluation consists of several standardized tests, but the manuscript does not indicate the approximate duration required for each assessment or the total expected testing time. Providing estimated administration times would be helpful for evaluating feasibility and participant burden. Additionally, clarification on whether the order of cognitive tests is fixed or randomized would be useful. In older adults, accumulated fatigue could influence performance and increase variability, and noting any mitigation strategy (e.g., breaks, counterbalancing) would strengthen the protocol description.	Thank you for this valuable suggestion to enhance feasibility and reproducibility reporting. We have added the following details to the "Baseline cognitive assessment":<br><br>The battery follows a fixed administration order to avoid interference effects and respect normative protocols: (1) ACE-R, (2) TAVEC, (3) ECog, (4) GDS (during TAVEC 20-min delay if needed), (5) TMT A/B, (6) Rey Complex Figure Test (20-min delay between copy/immediate and delayed recall, during which Verbal Fluency Test may be completed if pending), (7) Zoo Map Test. Estimated total duration: 90-120 minutes, varying by participant performance and including breaks as needed.<br><br>To mitigate fatigue in older adults, breaks are offered whenever requested by the participant or observed by the examiner (reduced alertness, motor slowing). If excessive fatigue prevents completion, the session is paused or rescheduled within 1 week maximum.

Reviewer 3:

Comments	Response
Abstract.<br>What does the word “analytical” signify?. “A cross-sectional, analytical, double-blind study is being conducted comparing 30 young adults (18–39 years) and 30 older adults (≥65 years).”	Thank you for this clarifying question. The term "analytical" has been removed along with "double-blind" as addressed in our response to Editor Comment 1 and Reviewer 1 (Abstract).<br><br>The Abstract now reads: "A cross-sectional, laboratory-based observational study is being conducted in a single session, in which participants are exposed to emotional stressors (high-arousal images) and their immediate neuromuscular and balance responses are recorded, comparing 30 young adults (18–39 years) and 30 older adults (≥65 years).

	This revision eliminates both terms while maintaining the cross-sectional comparison between age groups, as already revised in tracked changes.
Page 7 of the pdf document:<br><br>“No datasets were generated or analysed during the current study. All relevant data from this study will be made available upon study completion”. Please elaborate. How can you conduct a study without generating data? And please specify how the data will be made available.	Thank you for this important question. The statement "No datasets were generated or analysed during the current study" is the standard PLOS ONE wording for study protocols, as this manuscript describes methods rather than results (first participant enrolled Nov 27, 2024).<br><br>We have revised for clarity: "No datasets were generated or analysed during the current study, as this is a protocol manuscript. All de-identified raw data (EMG, pressure mat, IMU, HRV, cognitive/physical assessments) from the completed study will be made publicly available via ClinicalTrials.gov (NCT06682754) upon study completion, in line with open science principles."<br><br>This change appears in tracked changes.
Lines 157-183 Please include more information on the suspected effects of stress on the results.	Thank you for this constructive comment. We have expanded the description immediately preceding the general and specific objectives to include the expected physiological effects of emotional stress on postural and neuromuscular control, based on the reviewed literature and the attached ethics files (CEI Protocol). This clarifies the underlying mechanistic hypotheses that motivate the study objectives.<br><br>It is expected that high-arousal IAPS images will generate autonomic nervous system activation, increasing heart rate variability, possibly respiratory rate, and feelings of unease (measured with a VAS scale from 0 to 10). This may cause alterations in muscle activation, such as increased electrical activity in key muscles (tibialis anterior, gluteus medius, etc.) detected by electromyography; greater sway in ground pressure and displacements with some instability during tasks like standing up, sitting down, walking, or pouring water. In older adults, these effects could exacerbate fall risk. It is expected that high-arousal IAPS images will generate autonomic nervous system

	activation, increasing heart rate variability, possibly respiratory rate, and feelings of unease (measured with a VAS scale from 0 to 10). This may cause alterations in muscle activation, such as increased electrical activity in key muscles (tibialis anterior, gluteus medius, etc.) detected by electromyography; greater sway in ground pressure and displacements with some instability during tasks like standing up, sitting down, walking, or pouring water. In older adults, these effects could exacerbate fall risk.
Lines 157-183: I do not understand how the many tests of cognitive and physical ability relate to the research aims. Please explain.	Thank you for requesting clarification. The baseline cognitive and physical assessments characterize participants by frailty/cognitive status, enabling subgroup analyses to determine if stress-induced balance changes vary by vulnerability level.<br><br>These subgroup analyses are explained in detail in the "Statistical Analysis" section.
Lines 210-214:I note that being deaf or visually impaired is not a contraindication to participation in the study. How do you intend to handle these participants?	Thank you for this important observation. This concern was already addressed in our response to Reviewer 1. We clarified that inclusion requires ability to understand spoken and written Spanish sufficiently to follow the study information and instructions, and corrected vision for IAPS tasks, ensuring deaf or visually impaired participants can only be included if they can safely complete all study procedures with their usual assistive devices.
Line 214: Please supply more information on the consent procedure. You state: "Firstly, participants read the information sheet and sign the Informed Consent form. Following this, baseline cognitive and physical assessments are carried out (Fig. 2)." Are the participants verbally informed about the study? Are they able to take the time to consider participation? Can they ask questions? Consult	Thank you for this important question regarding the consent procedure. Participants receive both verbal and written information about the study. Researchers explain the study verbally, answer all questions, allow time for consideration, and encourage consultation with family or others. No consequences arise from declining or withdrawing participation. We have now detailed this process in the manuscript and attached the information sheet as Supplementary Information.

with others?	
Line 244, Fig 2. The test procedure is to view the low arousal pictures first, then the high-arousal pictures. Did you consider randomizing the order? Why not? Please explain. Please supply some information about typical time spent in the different parts of the experiment.	Thank you for this excellent question. Fixed order (low- then high-arousal) was deliberately chosen to mimic real-life stress exposure where emotional arousal typically follows neutral states, maximizing ecological validity. Randomization was considered but rejected as it risks carryover effects (high-arousal contamination of low-arousal condition) in vulnerable older adults.<br><br>Approximate mean times for each procedure have been added to Fig. 2.
Lines 445-460 Do you have a more detailed statistical analysis plan? Is it possible to make it part of the paper? The experiment includes both a data modelling part and an statistical analysis plan, how do these two parts fit together?	We thank the reviewer for their comment. Following their recommendations, the statistical analysis plan has been detailed in greater depth in the "Statistical Analysis" section within the "Materials and methods" section.<br><br>It should also be noted that this is a broad project with three main objectives. Data modelling responds to the third main objective of the study, which is to verify the potential of sensors to apply AI and data mining algorithms. On the other hand, statistical analysis responds to the first and second main objectives, which are to evaluate postural control alterations under emotional stress in young and older adults and to determine whether these oscillations increase the risk of falls in older adults. To clarify these issues, this information has been added to the opening paragraphs of the "Data modelling" and "Statistical Analysis" sections within the "Materials and methods" section.
Lines 215-219: Sample size calcutions. Please explain this in more detail. What statistical analysis is to be used? What are the estimated averages and variability of the different assessments. Did you do sample size calculations for each assessment separately?	We are very grateful to all reviewers for their comments on the sample size calculation. In order to take this suggestion and the recommendations of reviewers 1 and 2 into account, this calculation has been explained in greater detail in the 'Participants' section.<br><br>In this regard, it should be noted that this study is part of a broader project in which one of the main objectives is to compare alterations in balance, center of

	mass displacement and muscle activation induced by emotional stressors in young and older adults. Given that several primary outcome variables are analyzed, the sample size was calculated based on the effect size reported in previous studies that have addressed the impact of stress on balance in these populations.
Lines 340-353: Wil the EMG data be collected during physical test? The SPPB, Mini-BESTtest with TUG, and the Functional Reach Test?	Thank you for this important clarification question. No EMG data are collected during baseline physical tests (SPPB, Mini-BESTest with TUG, FRT). These assessments characterize participant frailty/cognitive status at study entry. EMG recording begins only during motor task testing (t1 low-arousal baseline, t2 high-arousal experimental), capturing stress-induced neuromuscular changes across 7 bilateral muscles per SENIAM guidelines. This distinction is now clearly stated in the Outcome measurements section.
Lines 342-367: Is it necessary to include information on software version of the Delsys Trigno, Fitness MAT DEV KIT, WT9011DCL devices and the Hexoskin Pro vest? Can you please describe how data is handled by these commercial devices? Is any data transmitted or passed through remote locations? Are any of these locations outside the European Union? How does the study comply with GDPR?	Thank you for this important question regarding device specifications and data handling. Software versions are included as they specify configuration options critical for reproducibility. All commercial devices (Delsys Trigno, Fitness MAT DEV KIT, WT9011DCL) operate in offline mode - data collected locally via wireless to UAH computers, with no remote transmission during acquisition. After acquisition, there is one device requiring special processing: Hexoskin data are stored on the Hexoskin server (AWS data centres, US West/East Coast). Some processing (HRV calculation) occurs there, as Hexoskin offers no EU-exclusive server option. However, local storage on computer (no server sync) is available, though this skips Hexoskin HRV processing.
Lines 396-397: Please describe the potential harm to the participants if sensitive data are compromised. Please also describe the potential emotional harm of viewing disturbing images.	Thank you for this important question regarding participant safety. Sensitive data compromise: Minimal risk due to pseudonymization (unique codes from outset), local storage (UAH OneDrive private folder/researcher access only; physical data under lock/key), and GDPR compliance - no identifiable personal data collected.

	Emotional harm from IAPS images: Transient mild discomfort possible from occasionally unpleasant images.<br><br>The statement “The measurement of variables and intervention of this study involves no risk, harm, or discomfort." has been replaced with a comprehensive risk description addressing data compromise and emotional effects from IAPS images.
Lines 404-405: Please be specific about what you mean by experiment? Is this the period from the consent form is signed, until the participants leave the laboratory?	Thank you to the reviewer for pointing this out. To improve clarity and be more specific, “During the experiment” has been changed to “During the baseline test (t1) and the experimental test (t2)” in the ‘Procedure’ section.
Lines 430-444. I am not competent to assess the paragraph on data modelling	Thank you for your honest assessment and for reviewing areas outside your expertise. The data modeling paragraph describes exploratory analyses using AI/ML techniques (cross-validation, performance metrics like AUC/precision-recall) on pseudonymized sensor data - positioned as secondary to primary statistical analyses (ANOVA on EMG RMS changes). We believe no changes are needed as this transparency aligns with PLOS ONE protocol guidelines for exploratory.
Lines 519-520: “The study of all the issues addressed in the DEPIE project will make it possible to take action before stressful situations arise,” This is a strong statement and I do not consider et supported by your study design. Please reconsider the sentence.	Thank you for this perceptive comment. We agree that the original statement is overly strong and not precisely supported by our cross-sectional laboratory design, which establishes stress-induced neuromuscular mechanisms rather than real-time clinical interventions.<br><br>The study of all issues addressed in the DEPIE project will enable action in response to stressful situations and help prevent potential falls or identify fall risk faced by individuals exposed to emotional stressors, allowing identification of underlying mechanisms to propose more appropriate interventions. We have revised the wording in the Conclusions section.

Lines 642-643, reference number 27. Please use standard formatting of references. I am not able to assess references in Spanish.	Thank you to the reviewer for your comment.<br><br>Reference number 27 has been revised in accordance with the journal's standard formatting references, and the name of the cited document has been translated into English.<br><br>Likewise, the style of all references has been revised.
Lines 658-660. Please use standard formatting of references. “Hermens HJ, Freriks B, Merletti R, Stegeman D, Blok J, Rau G, et al. European Recommendations for Surface ElectroMyoGraphy Results of the SENIAM project. Roessingh research and development. 1999; 8(2).” Are you referring to “Hermens, Hermie J., et al. "European recommendations for surface electromyography." Roessingh research and development 8.2 (1999): 13-54.»	Thank you to the reviewer for pointing this out. Indeed, we were referring to that publication. We have revised the citation in accordance with the formatting guidelines of PLOS ONE and the missing data.

We hope that all questions have been answered satisfactorily and we remain attentive to any questions that need to be answered.

On behalf of the team,

Yours sincerely.
